# Anatomical study of the innervation of the tibialis anterior muscle and its relationship with myofascial trigger points

**DOI:** 10.1590/acb414326

**Published:** 2026-07-24

**Authors:** Jéssica Bevilaqua Congio, César Rocha de Alencar, Flávio Carneiro Hojaij, Mauro Figueiredo Carvalho de Andrade, Alfredo Luiz Jacomo, Flávia Emi Akamatsu

**Affiliations:** 1 Universidade de São Paulo – Faculty of Medicine – Department of Surgery – São Paulo (SP) – Brazil.

**Keywords:** Anatomy, Trigger Points, Muscles

## Abstract

**Purpose:**

Myofascial trigger points (MTPs) in the tibialis anterior muscle (TAM) are located in the upper third of the muscle and can be activated by various pathologies such as sprains, fractures, and muscle overload. Although the pathophysiology of MTPs is unclear, they often coincide with motor plates in the innervation zone. This study aimed to describe the innervation of the deep fibular nerve (DFN) into the TAM and relate it to clinically described MTPs.

**Methods:**

The TAMs of 12 cadavers were dissected from their origins to observe the exact point at which the DFN penetrates the muscle belly. Entry points were mapped by dividing the muscle into four areas. Statistical analysis was performed using a Poisson marginal distribution and identity linkage function, followed by Bonferroni multiple comparisons. Statistical analysis included summary measures, group comparisons using Student and Mann–Whitney’s tests, and Pearson and Spearman’s correlations. Statistical significance was set at *p* < 0.05.

**Results:**

Quadrants I and II had significantly more DFN branches than quadrants III and IV (*p* < 0.001), whereas quadrants I *versus* II and areas III *versus* IV showed no significant differences (*p* < 0.05).

**Conclusion:**

Consistent with the clinical literature, the branches of the DFN in the TAM correspond to the described areas of the MTPs. The anatomical relationships between MTPs may help explain the pathophysiology of various disorders and provide rational treatment options.

## Introduction

**Figure 1:**
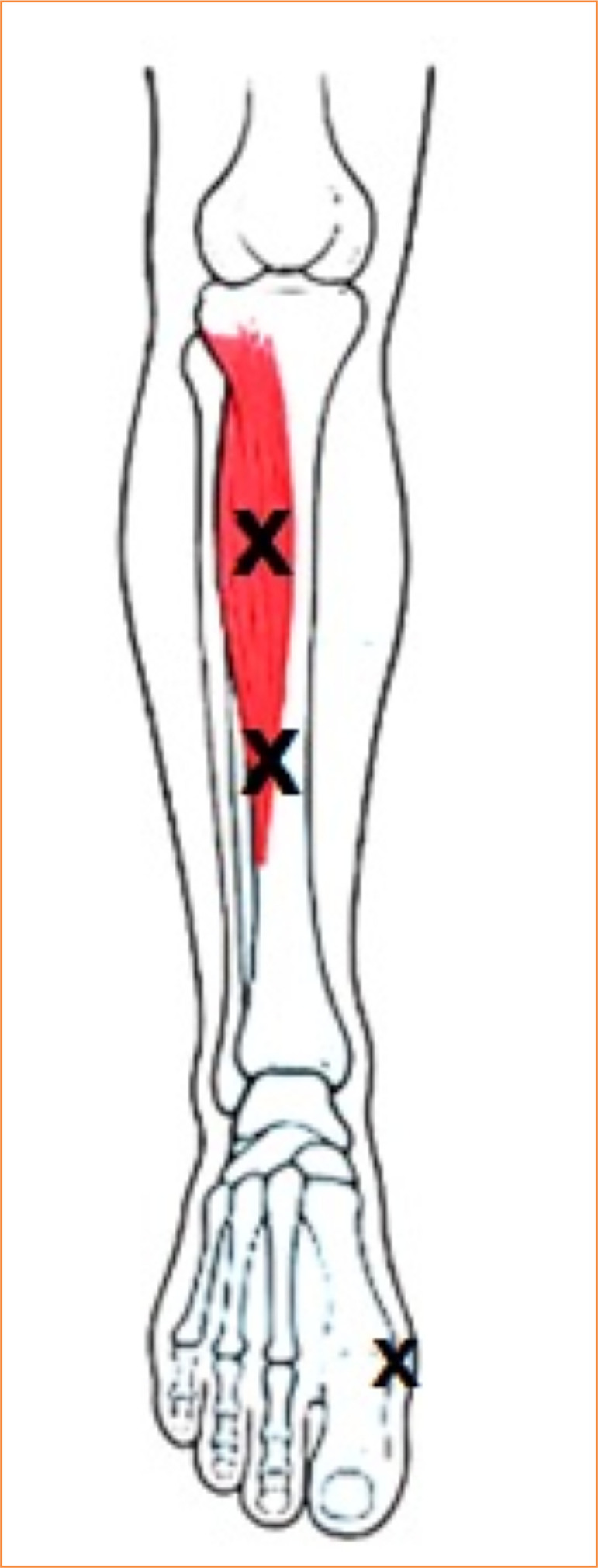
Myofascial trigger points (x) in the tibialis anterior muscle.

Source: Elaborated by the author Jéssica Bevilaqua Congio based on Simons et al.^2^.

Myofascial trigger points (MTPs) in the tibialis anterior muscle (TAM) are characterized by localized pain that increases with stretching and by the presence of trigger points (TPs) in the muscle belly^1^, particularly in the upper third of the TAM^2^. Referred pain and tenderness caused by TPs in TAM often present on the anteromedial aspect of the ankle and dorsal and medial surfaces of the hallux^2^ (Fig. 1). Changes in TAM activation caused by conditions, such as walking on uneven surfaces, can precipitate myofascial dysfunction^3-5^, leading to chronic pain^6,7^. Chronic leg pain is common among both professional and amateur athletes^8^.

The TAM, a superficial and easily palpable muscle innervated by the deep fibular nerve (DFN, L4 and L5), plays a fundamental role in ankle dorsiflexion and foot inversion^9^. According to Standring^9^, the TAM originates from the lateral condyle and the proximal half to two-thirds of the lateral aspect of the tibial diaphysis, from the anterior aspect adjacent to the interosseous membrane, from the deep part of the deep fascia, from the intermuscular septum, and between it and the extensor digitorum longus. The TAM descends vertically and ends in a tendon on the anterior surface of the lower third of the leg. It inserts into the medial and inferior surfaces of the medial cuneiform bone and the adjacent part of the base of the first metatarsal, at the base of the proximal phalanx of the hallux, and in the extensor retinaculum^9^.

Chronic myofascial pain syndrome (MPS) is the most frequent cause of skeletal muscle pain, with an estimated prevalence of 85% in the general population^10^. Clinically, MPS is associated with TPs, which include both sensory (nociceptors that cause pain) and motor components. Excess acetylcholine release at dysfunctional motor endplates leads to sustained muscle contractions, palpable taut bands, hypersensitive nodules, and limited movement^2,11,12^. Each muscle exhibits a characteristic referred pain pattern based on MTP location.

Anatomical studies have demonstrated overlap between nerve entry zones and clinically described MTPs^2,13^. Xie et al.^14^ found that intramuscular nerve distribution in the trapezius closely corresponds to MTP sites, suggesting that dysfunction in motor endplate regions may underlie trigger point development. Other studies have similarly reported correlations between clinically identified MTPs and specific anatomical regions^15,16^.

Recent imaging studies using ultrasound^17^ and magnetic resonance imaging (MRI)^18^ have confirmed the presence of MTPs and taut bands, localized areas of increased muscle stiffness associated with local constriction caused by taut bands or inflammation or autonomic changes associated with altered perfusion, inflammation, and autonomic change. The resulting ischemia and hypoxia may promote the release of chemicals that irritate and activate peripheral nociceptors, thereby causing pain. These findings support Simons’ model, suggesting that excessive acetylcholine release triggers abnormal contractions within taut bands, leading to ischemia and activation of peripheral nociceptors^13,19^. Although emerging diagnostic tools, such as ultrasound, vibration sonoelastography, muscle pain detection devices, Doppler flow studies, and magnetic resonance elastography have been explored for objective diagnosis, further research is needed to determine their effectiveness^20^.

The true prevalence of myofascial pain (MP) remains uncertain because of the absence of precise diagnostic criteria^21^. Moreover, no studies have investigated the relationship between DFN entry points and DFN with MTP distribution in the TAM. Understanding the innervation patterns of the TAM and their relationship with MTPs may clarify the pathophysiology of MPS and guide the development of more effective clinical and surgical interventions in the TAM.

## Methods

### Ethical aspects

This study was approved by the Research Ethics Committee of the Faculty of Medicine, Universidade de São Paulo, Brazil (research protocol number: 5.712.901).

### Anatomical technique

This study used the method described by Akamatsu et al.^22^, and the description of the methods partly reproduces their wording^22^.

**Figure 2:**
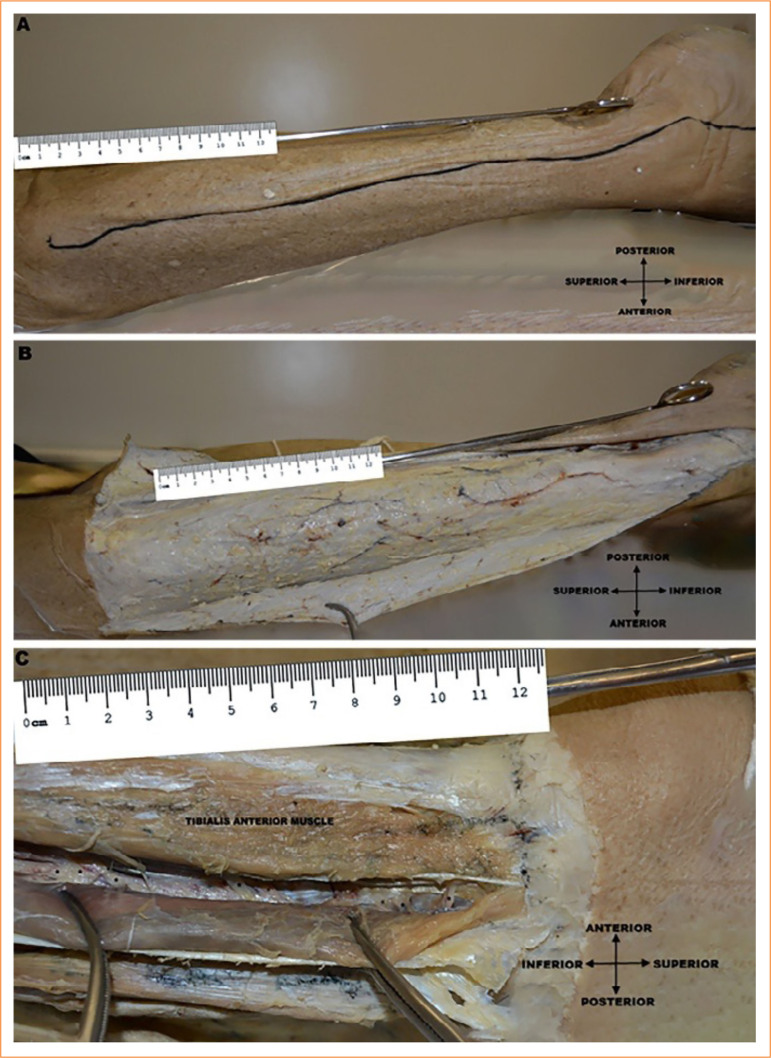
Dissection steps. (a and b) Left lower limb in the prone position. (a) Line marking the incision site. (b) Skin folded back to expose the subcutaneous connective tissue. (c) Subcutaneous connective tissue folded back to expose the left tibialis anterior muscle and nerves in the supine position.

Source: Elaborated by Flávia Emi Akamatsu Jacomo.

**Figure 3:**
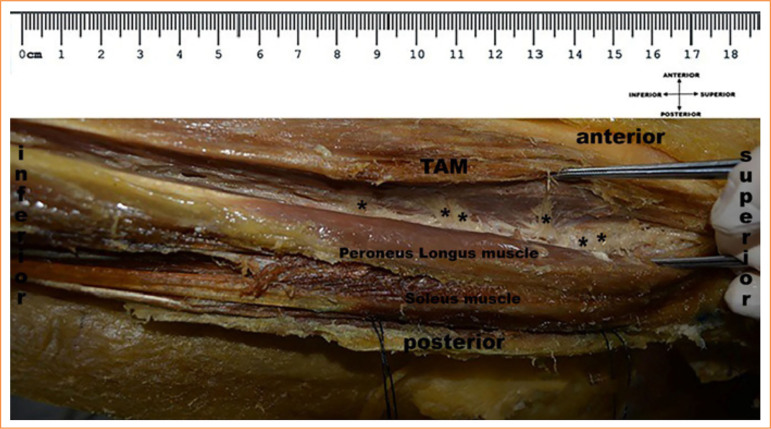
Left lower leg in the supine position. The nerve entry points into the left tibialis anterior muscle are exposed, showing innervation by the deep fibular nerve.

Twenty TAMs from 12 adult human cadavers (eight females and four males) donated to the Human Structural Topography Discipline of the Department of Surgery of the Faculty of Medicine were dissected. The cadavers were fixed in 4% phenolic acid and 0.5% formaldehyde solution. Only specimens with no signs of previous surgical manipulation or other visible abnormalities in the regions of interest were included. Dissection was performed until the DFN branches, and their entry points into the TAM were exposed. The specimens were placed in the prone position to facilitate the location of the common fibular nerve in the popliteal fossa. The skin was then incised from the popliteal fossa to the ankle, using the medial malleolus as a limit. The skin was reflected with the subcutaneous cellular tissue and fascia to expose the muscles. When a DFN was found, it was followed until the TAM was reached. The entry points of the DFN were mapped (Figs. 2 and 3).

*Innervation. Source: Elaborated by Flávia Emi Akamatsu Jacomo.

### Tibialis anterior muscle measurements and quadrant delimitation

**Figure 4:**
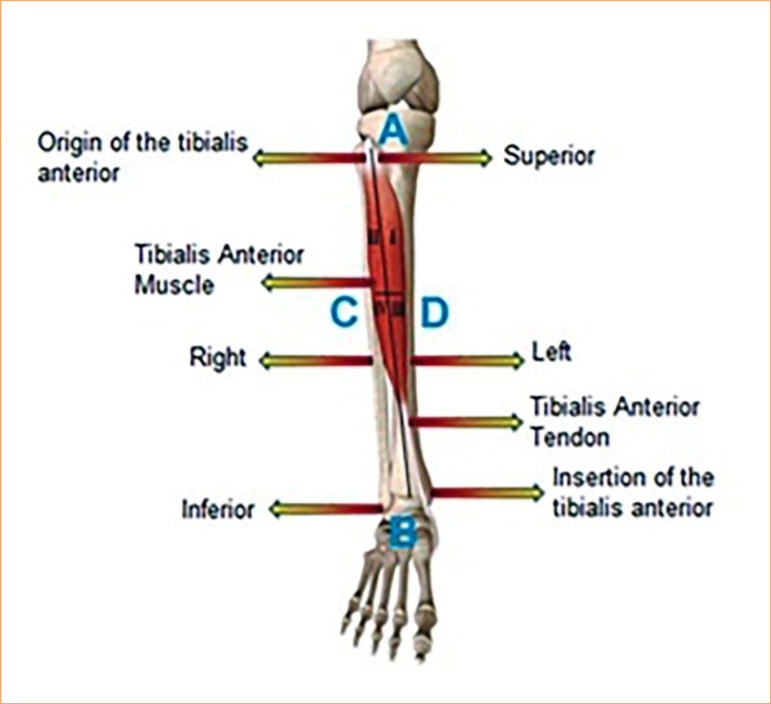
Measurements of A, B, C, and D and the quadrants used in the present study.

Source: Elaborated by Jéssica Bevilaqua Congio.

The Akamatsu method was adopted, with some modifications. After dissection, the TAMs were measured using digital calipers accurate to 0.1 cm. The longitudinal length (AB), defined as the largest muscular dimension, extended from the tibial tuberosity to its tendon at the level of the medial malleolus. The transverse line (CD) was defined at the midpoint of the longitudinal axis (AB) of the muscle. Using these reference dimensions, the entry points of the DFN were mapped, and the muscle was divided into four quadrants: quadrants I and II, in the upper region, and quadrants III and IV, in the lower region, oriented from the medial to the lateral direction (Fig. 4).

The entry points of the DFN into the TAM in relation to the middle transverse and longitudinal measurements were determined based on AB and CD, and a plane was delimited on the abscissa and ordinate, respectively.

The relative values of the penetration point in relation to the mean transverse and longitudinal dimensions were calculated, as these values tend to vary depending on the muscle size. Thus, these dimensions constituted 100% of the muscle size, with the muscle insertion values making a small contribution. The intersection of the axes was defined as the origin (zero point). The superior–medial quadrant had positive abscissa and ordinate values; the inferior–lateral quadrant had positive abscissa and negative ordinate values; the superior–lateral quadrant had negative abscissa and positive ordinate values; and the inferior–medial quadrant had negative abscissa and ordinate values (Fig. 4).

Four quadrants (I–IV) were defined to group the data into categories to facilitate clinical correlations, with the middle transverse line separating the upper and lower areas. Quadrants I and II corresponded to the mediolateral superior area, while quadrants III and IV corresponded to the mediolateral inferior areas (Fig. 4).

**Figure 5:**
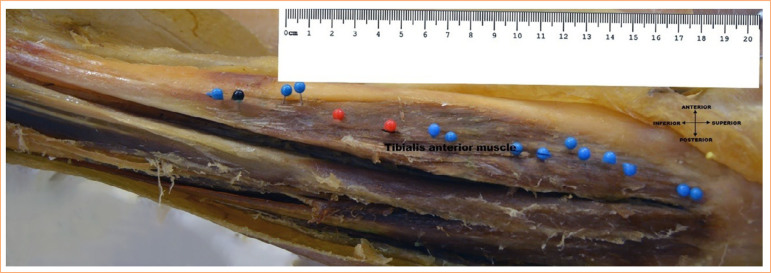
Left lower limb in the supine position showing the left tibialis anterior muscle with pins marking the entry points of the deep fibular nerve into the muscle belly.

Source: Elaborated by Flávia Emi Akamatsu Jacomo.

**Figure 6:**
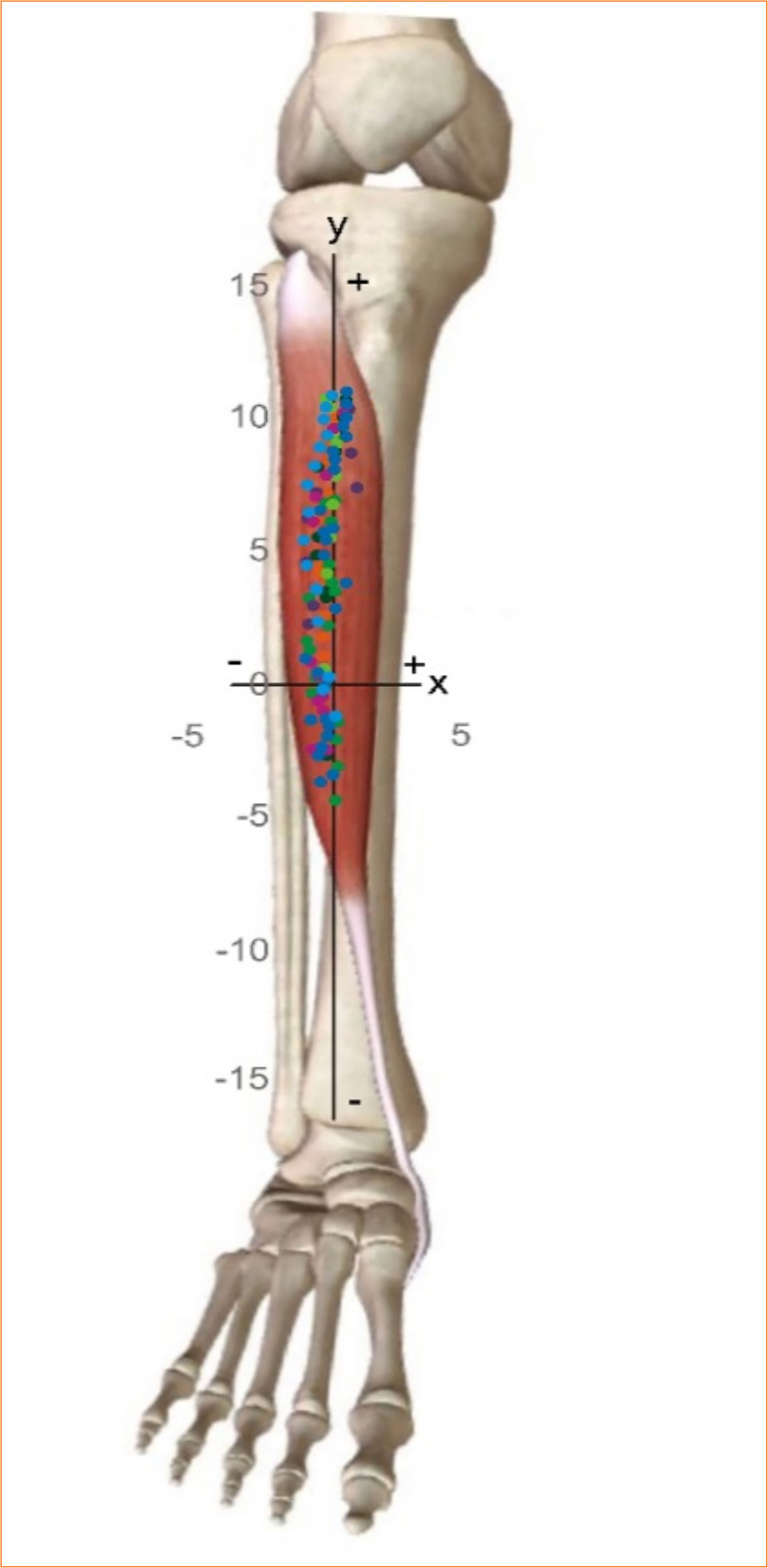
Entry points of the deep fibular nerve into the tibialis anterior muscle.

Source: Elaborated by the author Jéssica Bevilaqua Congio.

The entry points of the DFN branches into the muscle were marked using colored pins (Fig. 5). Photographs of all specimens were acquired (Nikon D52). The locations of the entry points in relation to the mean longitudinal and transverse axes were measured via a simple division of the values and classified into Areas I–IV (Fig. 6).

### Statistical analysis

#### Sample calculation

The sample size was estimated using data from the first five cadavers (10 muscles) examined in the pilot study, which showed a mean difference of 3.6 points (standard deviation = 3) between quadrants I and IV. To detect this difference with 80% power and 95% confidence interval (95%CI), a minimum of 16 muscles were required, assuming a two-tailed test^23^.

Summary measurements (mean, standard deviation, median, minimum, and maximum) were used to describe the quantitative characteristics of the cadavers, whereas absolute and relative frequencies were used to describe the qualitative characteristics.

### Analysis of results

Muscle characteristics were described according to sex using summary measures and compared between sexes using unpaired Student’s t-tests^24^. The total numbers of nerve entry points per muscle on each side were also summarized according to sex and compared by the Mann–Whitney’s test^24^. Pearson’s correlation coefficients were calculated to assess the relationships between the quantitative cadaver characteristics and the muscle measurements, whereas Spearman’s correlations were used to investigate the associations involving the total number of points per side.

Muscle measurements and total points were described according to laterality and compared between sides using paired Student’s t-test and unpaired Wilcoxon signed-rank test, respectively^24^.

The number of nerve entry points in each muscle was analyzed by quadrant and compared between sides and quadrants using generalized estimating equations with an interchangeable correlation matrix between sides and quadrants. The model assumed a marginal Poisson distribution with an identity link function^25^. Bonferroni-adjusted multiple comparisons^24^ were subsequently applied to identify specific differences among quadrants.

All data are presented as means and standard deviation. Statistical analyses were performed using IBM Statistical Package for the Social Sciences for Windows, version 26.0 (IBM Corp., Armonk, NY, United States of America), and data were tabulated using Microsoft Excel. Significance was set at 5%.

## Results

The age of the cadavers ranged from 60 to 94 years old (mean: 78.4). The approximate heights ranged from 1.5 to 1.8 m (mean = 1.65). The weight and body mass index (BMI) ranged from 40 to 85 kg (mean = 62.5) and 14.7 to 33.2 kg/m^2^ (mean = 23.95), respectively. All 12 cadavers were of White (Caucasian) descent (Table 1). Data were not obtained from all cadavers, as four were incomplete. Therefore, the analysis included eight participants.

**Table 1:** Cadaver characteristics.

Variable	Description
**Age (years old)**	**(n = 12)**
Mean ± SD	78.4 ± 12,8
Median (p25; p75)	79 (66; 91)
**Sex**	**(n = 12)**
Female	8 (66.7)
Male	4 (33.3)
**Body mass index (kg/m** ^2^ **)**	**(n = 8)**
Mean ± SD	21.6 ± 5.8
Median (p25; p75)	21.7 (16.5; 24.1)

SD: standard deviation. Source: Elaborated by the authors.

The measurements and number of DFN entry points per side did not differ significantly between the sexes (*p* > 0.05) (Table 2).

**Table 2:** Comparison of tibialis anterior muscle measurements and total number of entry points according to sex<tfn href="tfn01">*</tfn>.

Variable	Sex		*p* -value
	Female	Male	
**Right TAM A-B (cm)**			0.119
Mean ± SD	28.8 ± 3.4	33.7 ± 2.5	
Median (p25; p75)	30 (25; &)	34 (31; &)	
**Left TAM A-B (cm)**			0.270
Mean ± SD	30.7 ± 2.2	32.7 ± 2.1	
Median (p25; p75)	30.8 (28.5; 32.7)	32 (31; &)	
**Right TAM C-D (cm)**			0.670
Mean ± SD	1.7 ± 1.2	2 ± 0.5	
Median (p25; p75)	1 (1; &)	2 (1.5; &)	
**Left TAM C-D (cm)**			0.650
Mean ± SD	1.8 ± 1.2	2.2 ± 1	
Median (p25; p75)	1.3 (1; 3)	2.5 (1; &)	
**Entry points – Right side**			0.476£
Mean ± SD	12.8 ± 4.2	15 ± 5.8	
Median (p25; p75)	11.5 (10.5; 15)	15 (9,.5; 20.5)	
**Entry points – Left side**			0.154£
Mean ± SD	10.4 ± 4	15 ± 5.2	
Median (p25; p75)	9 (7.3; 12)	13.5 (11; 20.5)	

* Paired Student’s t-test; £: unpaired Mann–Whitney’s U test; &: data could not be calculated; SD: standard deviation; TAM: tibialis anterior muscle.

Source: Elaborated by the authors.

The TAM measurements and total number of points did not differ significantly between the right and left sides (*p* > 0.05). Paired Student’s t-test was used for muscle measurements, whereas the unpaired Wilcoxon test was used to compare the point totals between the sides (Table 3).

**Table 3:** Comparisons of tibialis anterior muscle measurements and total number of entry points per side<tfn href="tfn02">#</tfn>.

Variable	Side		*p* -value
	Right	Left	
**TAM A-B (cm)**			0.586
Mean ± SD	31.3 ± 3.8	31.5 ± 2.2	
Median (p25; p75)	31.3 (28.8; 34.5)	31.6 (30; 33)	
**TAM C-D (cm)**			0.750
Mean ± SD	1.8 ± 0.8	1.9 ± 1.1	
Median (p25; p75)	1.8 (1; 2.6)	1.5 (1; 3)	
**TAM Points**			0.341*
Mean ± SD	13.7 ± 4.7	11.9 ± 4.8	
Median (p25; p75)	12.5 (10.5; 17.3)	11 (8; 15)	

# Paired t-test; SD: standard deviation;

* unpaired Wilcoxon test;

TAM: tibialis anterior muscle.

Source: Elaborated by the authors.

The number of points in the left tibia was significantly inversely correlated with cadaver age (r = -0.736, *p* = 0.006). As age increased, the number of points in the left TAM tended to decrease. Pearson’s correlations were used for the continuous variables, while Spearman’s correlations were used for the total number of points (Table 4).

**Table 4:** Correlations between cadaver quantitative parameters and tibialis anterior muscle measurements and total points#.

Correlation		Age (years old)	Body mass index (kg/m^2^)
Right TAM A-B (cm)	r	-0.634	-0.861
	p	0.176	0.061
Left TAM A-B (cm)	r	-0.471	-0.629
	p	0.286	0.181
Right TAM C-D (cm)	r	0.131	-0.760
	p	0.805	0.136
Left TAM C-D (cm)	r	0.059	-0.385
	p	0.899	0.452
Right TAM Points*	r	-0.196	-0.086
	p	0.588	0.872
Left TAM Points*	r	-0.736	0.169
	p	**0.006**	0.690

# Pearson’s correlation;

* Spearman’s correlation;

r: correlation coefficient, with values ranging from -1 to 1, where -1 indicates an inverse correlation and 1 indicates a direct correlation; p: assessment of the statistical significance; TAM: tibialis anterior muscle.

Source: Elaborated by the authors.

The number of points differed significantly between the quadrants of the TAM (*p* < 0.001), with a greater number of points observed in quadrants I and II compared with quadrants III and IV (Table 5).

**–**

**Table 5:** Description of the number of points in the tibialis anterior muscle by quadrants and comparison<tfn href="tfn01">*</tfn>.

Variable	Quadrant				*p* -value
	I	II	III	IV	
**TAM Points**					**< 0.001**
Mean ± SD	4.9 ± 4.4	4.3 ± 3.,1	1.5 ± 1.5	2.1 ± 1,.9	
Median (p25; p75)	4.5 (1; 8.5)	4 (2; 6)	1.5 (0; 3)	1.5 (0.8; 4)	

* Generalized estimating equation with Poisson’s distribution and identity link function, assuming an exchangeable correlation matrix between sides and quadrants;

TAM: tibialis anterior muscle; SD: standard deviation.

Source: Elaborated by the authors.

**Figure 7:**
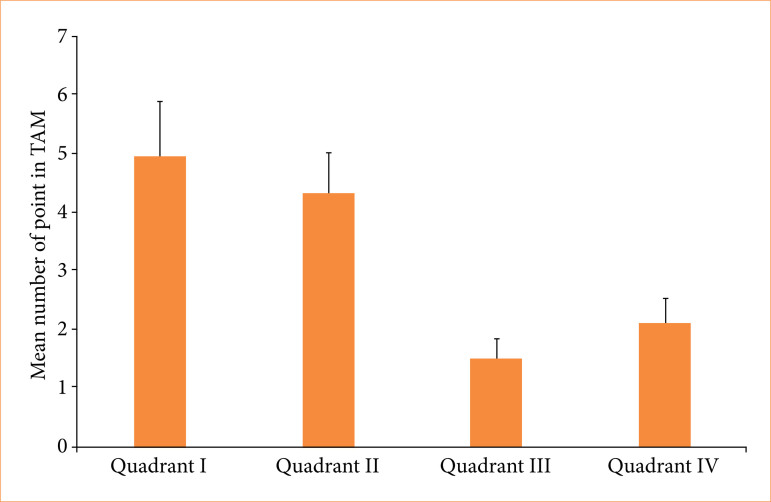
Mean numbers of points in the TAM according to quadrant, with respective standard errors. The number of points differs significantly between quadrants I and II and quadrants III and IV, with a higher concentration of points in the upper quadrants (I and II) than in the lower quadrants (III and IV). Quadrants III and IV show no significant differences.

The average differences in the number of points did not differ significantly for quadrant I *versus* II and quadrant III *versus* IV (*p* > 0.05), while the greater number of points was significantly higher in quadrants I and II than in quadrants III and IV (*p* < 0.001) (Table 6, Fig. 7).

**Table 6:** Comparisons of the number of nerve entry points in the tibialis anterior muscle between quadrants*.

Quadrant comparison	Mean difference	Standard error	*p* -value	95%CI	
				Inferior	Superior
I and II	0.59	0.64	> 0.999	-1.10	2.29
I and III	3.36	0.54	**< 0.001**	1.95	4.78
I and IV	2.77	0.56	**< 0.001**	1.29	4.25
II and III	2.77	0.51	**< 0.001**	1.42	4.12
II and IV	2.18	0.54	**< 0.001**	0.77	3.60
III and IV	-0.59	0.40	0.856	-1.65	0.47

* Bonferroni multiple comparison tests;

TAM: tibialis anterior muscle; 95%CI: 95% confidence interval.

Source: Elaborated by the authors.

TAM: tibialis anterior muscle. Source: Elaborated by the authors.

## Discussion

Although no previous studies have specifically investigated the distribution of the DFN and the location of MTPs in the TAM, numerous reports have suggested a relationship between muscle innervation and TPs formation in other muscles, including the trapezius, gluteus maximus, abductor hallucis, masseter, and temporalis^22,26-29^. These studies collectively indicate an anatomical overlap between regions of dense nerve branching and MTP locations.

Descriptions of MTP distribution have varied across studies. The present study adopted the characterization by Travell and Simons^13^, who localized MTPs to the superior third of the TAM, as more precise data for this muscle were previously unavailable. Consistent with other anatomical studies, our findings identified a concentration of DFN entry points in the initial branching region in which it reached the belly of the TAM. This finding corroborates classic studies by Duchenne^30^, Remak^31^, and Ziemssen^32^, who described the distribution of innervation in these muscles. In addition, our analysis revealed that the DFN entry points were predominantly located in the upper quadrants (I and II) of the TAM, an area that coincides with the regions described by Travell and Simons^13^ as MTPs. This distribution pattern may be related to the higher density of muscle fibers in these areas, which, according to Blaauw et al.^33^, is essential for fulfilling muscle functions such as ankle dorsiflexion.

Our results align with those of Yi et al.^34^, who mapped nerve distribution in the TAM and observed a region of intense arborization between 70 and 80% of the muscle length. Although our measured TAM length was longer than their report (31.3 *versus* 22.7 cm), this difference can be attributed to anatomical variations between populations of different ethnicities. Yi et al.^34^ studied an East Asian cohort, generally characterized by shorter stature and limb length.

We observed a negative correlation between age and the number of DFN entry points in the TAM. This finding is consistent with the results of Siddiqi et al.^35^, age-related declines in motor unit number and efficiency, leading to reductions in strength and control in frequently used muscles such as the TAM. Therefore, decreased nerve insertion may be related to a decline in muscle strength and motor control in the elderly, offering insight into the pathophysiology of myofascial syndromes in aging populations.

Our findings agree with the observations reported by Stålberg et al.^36^ and Happak et al.^37^, who highlighted the differences in the pattern of innervation between muscles in different regions of the body, suggesting that the functional specificity of muscles can influence the number of motor units and nerve distribution. The TAM, which is responsible for ankle dorsiflexion, exhibits muscle architecture and innervation closely related to its function, which reinforces the idea that MTPs may occur, in part, because of the nerve distribution in these areas of high functional demand. The DFN is present as a long branch parallel to the muscle, emitting smaller branches that enter the muscle and supply its area.

Our initial hypothesis that MTPs are associated with muscle innervation was corroborated by the finding that the entry points of the DFN topographically coincide with the MTPs described in the clinical literature. This pattern is consistent with the conclusions of Akamatsu et al.^22,26^, Wada et al.^27^, Pinheiro et al.^28^, and Garrido^29^, who reported overlaps between the points of nerve penetration and MTP locations in other muscles. This anatomical overlap strengthens the hypothesis that dysfunction in the motor plate zones is a causal factor in MTPs, especially when associated with mechanical or inflammatory stimuli. This correlation may offer new perspectives for the treatment of myofascial pain and provide an anatomical basis for the development of more effective therapeutic approaches.

Advanced diagnostic tools, such as ultrasonography^17^ and MRI^18^, have confirmed the presence of MTPs and taut bands, supporting the model proposed by Travell and Simons^13^, that MTPs are caused by excessive acetylcholine release, resulting in aberrant contractions in a taut band.

The differential diagnosis between fibromyalgia and MP syndrome, which share similar symptoms, identification of MTPs and understanding their correlation with innervation, can improve diagnostic accuracy and therapeutic targeting. As Chandola and Chakraborty^38^ noted, improved understanding of the neuromuscular anatomy and pathophysiology associated with myofascial pain allows detailed clinical assessments to distinguish between these conditions and implement appropriate treatments.

Finally, Akamatsu’s methodological framework, which has been widely validated in several studies^22,26-29^, was fundamental to this study, as it allowed us to accurately identify the entry points of the nerves and their relationship with MTPs. This suggests that detailed mapping of the intramuscular nerve innervation may not only broaden our understanding of myofascial pain but also provide valuable information for safer and more effective clinical practices. The spatial location of nerve entry points in muscles can inform diagnosis and treatment by improving the accuracy and time ratio of myofascial trigger point pain complaints. Methods such as acupuncture, shockwave therapy, local anesthetic injections, and dry needling have been used to alleviate symptoms associated with MTP abnormalities, with some authors reporting therapeutic benefits^39-45^.

Overall, the results of this study reinforced the hypothesis that nerve distribution within the TAM is closely related to MTP formation, with direct implications for the diagnosis and treatment of conditions associated with muscle pain, such as MPS.

### Study limitations

First, we were unable to form age- and race-based groupings because the cadavers were not accessible for selection. Additionally, because cadaveric analysis cannot replicate conditions in living individuals, it cannot establish direct correlations between MTPs and their physiological manifestations. Such correlations, which would provide greater insight into the pathophysiology and diagnosis of myofascial disorders, cannot be examined through dissection in a living person.

Moreover, the statistician determined the minimum sample size required to adequately represent the general population in accordance with the study’s objective; namely, to establish a connection between MTPs and DFN entry points, while accounting for the limited availability of cadaver donations in our country.

Finally, the sample calculation was based on a pilot analysis of the first five cadavers (10 muscles), in which the mean difference between quadrants I and IV for the tibial muscle was 3.6 points, with a standard deviation of 3 points. To detect this difference with 80% power and a 95%CI, the required sample size was estimated to be 16 muscles, assuming a two-tailed test^23^.

## Conclusion

This study described the entry points of the DFN into the belly of the TAM and established a relationship between these points and the MTPs described in the literature. The distribution of nerve branches in the TAM follows anatomical patterns that overlap with areas with the highest incidence of MTPs, reinforcing the hypothesis that these MTPs are closely linked to muscle innervation in MPS.

## Data Availability

The data will be available upon request.
